# An interactive tool for visualization of relationships between gene expression profiles

**DOI:** 10.1186/1471-2105-7-193

**Published:** 2006-04-06

**Authors:** Peter Ruzanov, Steven JM Jones

**Affiliations:** 1 Genome Sciences Centre, BC Cancer Research Centre, Ste 100-570 West 7th Ave Vancouver, BC V5Z 4S6, Canada

## Abstract

**Background:**

Application of phenetic methods to gene expression analysis proved to be a successful approach. Visualizing the results in a 3-dimentional space may further enhance these techniques.

**Results:**

We designed and built TreeBuilder3D, an interactive viewer for visualizing the hierarchical relationships between expression profiles such as SAGE libraries or microarrays. The program allows loading expression data as plain text files and visualizing the relative differences of the analyzed datasets in 3-dimensional space using various distance metrics.

**Conclusion:**

TreeBuilder3D provides a simple interface and has a small size. Written in Java, TreeBuilder3D is a platform-independent, open source application, which may be useful in analysis of large-scale gene expression data.

## Background

Development of high throughput transcription profiling techniques such as microarrays and SAGE [2] has made it possible to evaluate the expression levels of genes on a genome wide scale. The ability to look at expression profiles of different cellular states has the potential to provide knowledge about the mechanisms driving such important events as differentiation, aging and tumorogenesis. In addition to understanding transcription profiles of individual genes, the ability to visualize relationships between whole sets of gene expression data provides the means to infer higher-order relationships between the sampled biological conditions [8]. In this paper, we describe the design and usage of an interactive viewer, which builds hypothetical phenetic networks from SAGE expression data. These networks may help to associate analyzed SAGE libraries with some intermediate stages in the development of different cellular states or progress of disease, such as cancer. As an example, we have used our program to analyze publicly available SAGE data, but our application is also applicable to microarray data analysis.

## Implementation and results

Traditionally, hierarchical data are presented as a rooted or unrooted tree with bifurcating branches connecting different data points (nodes). Generally, only the distances along the edges of the tree are shown proportionally. In a non-reticulated tree, the distance between nodes that are not directly connected is not easily visualized or understood. Building a hierarchical tree benefits by operating in 3-dimensional space, as such 3-dimensional structure provides more details about the relationships between the analyzed datasets.

The algorithm of building phenetic networks or dendrograms, which we have used for our viewer, is generally applied in systematic biology. For systematic analysis, tree-building techniques are designed to order data in terms of phylogenetic relationships, from which evolutionary history can be inferred. In contrast, system analysis of SAGE and microarray data assumes that extraction of order in the relationships between different gene expression profiles and presents clues about biological processes and functions on molecular, cellular and tissue levels. Systematic approaches may allow improvement of diagnosis, disease classification, and prognosis, as well as assist in drug design, among other scientific and medical goals.

We have developed a graphical viewer for analysis of the hierarchical organization of cellular expression profiles, analyzed by SAGE or microarray experiments. The output diagram is a system of nodes, each of which represents a SAGE or microarray experiment, and the connections between nodes in the diagram. For each node, the relative position on the graph depends on the differences between its expression profile and the expression profiles of the other analyzed nodes. In order to generate the diagram, the user can choose a distance metric to generate the comparison. Our viewer currently offers five distance metrics, all having a different approach to the estimation of the distance between expression profiles. These metrics include Euclidean, Manhattan, Pearson and Pearson squared distances [4,5]. In addition, we have implemented a generic, 'quantitative' metric, which calculates distance as a ratio of the number of differently expressed genes to the number of similarly expressed genes (statistical significance of the difference in gene expression validated by Z-test as in [6])

**Figure 1:**
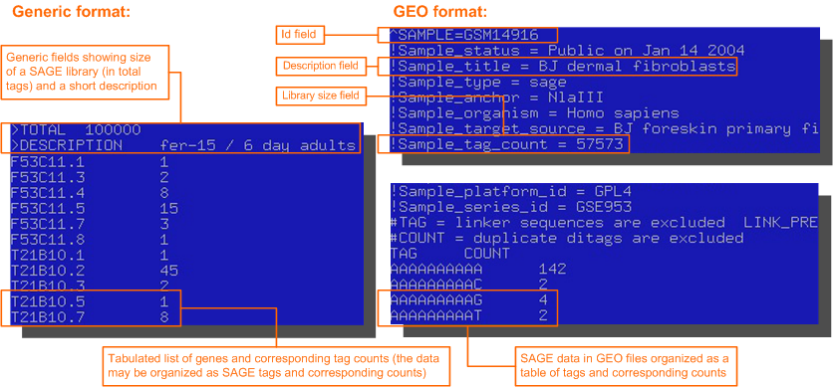
**Input formats**. **Left**: generic format, tab-delimited flat text file with (optional) fields "Total" and "Description", which may provide the application with information about total number of tags in a library and an alternative description (different from id string). The description becomes visible when user checks "Descr" box on the control panel. **Right**: data in GEO format. Highlighted are the fields, which are used for identification of the data-source and normalization of the tag counts. Also, it is possible to derive the short description from the field "Sample_title", which is analogous to the generic "Description" field.

The application loads the data from plain tab-delimited text files. Currently, we provide support for two formats – generic, tab-delimited with optional tags for description and total number of tags in a SAGE library and GEO (files of this type are downloadable from Gene Expression Omnibus website [7]). Format of compatible files is shown in Figure 1. The calculation of distances is performed via a pairwise analysis, so the distance value is calculated for each possible pair of SAGE or microarray experiments. In all five cases the values are normalized and put into an array, which is used to draw the graph. This array, or 'distance matrix' may be exported as a plain text file.

Our viewer automatically positions the analyzed nodes in 3D-space according to the calculated distances between them. In comparison to traditional phenetic techniques, mostly generating 2D-diagrams, this approach has several advantages. The constraints imposed by 2D-space are removed, so it is possible to get a more adequate snapshot of the system relationships between datasets. The nodes are connected, and by default it is possible to change the visibility for the connecting edges, adjusting the threshold for maximal length to be shown. In addition, in the default view mode the color of the edges reflects the tension, indicating if an edge is compressed or stretched. As the initially displayed length of an edge may be different from the calculated value for this connection, the user can estimate how accurate the lengths of the edges are is by looking at their color. TreeBuilder3D shows stretched edges in blue and compressed edges in red. As an alternative way to filter out insignificant (very distant) connections, our viewer can use a simple aggregation algorithm, analogous to Neighbor-joining method using arithmetic averages, described in [1]. After the 'distance matrix' is loaded into the viewer, nodes are placed randomly and the application starts to move nodes in order to keep the length of connecting edges proportional to their input values. The application's algorithm attempts to achieve the most relaxed state of the graph, which should give the most informative display of relationships between datasets, as at that point the displayed length of the edges should become proportional to the input distances. The relaxation algorithm checks one node at a time, making the adjustments for the distances between the current node and all the others. Eventually, after several cycles, diagram becomes stabilized (nodes stop moving, as the attracting and repelling forces balance each other). However, the absolute energetic minimum is rarely achieved – especially, with large number of data points. We tested the TreeBuilder3D, loading different number of SAGE libraries, and do not recommend opening more than 30 libraries, as it both degrades the performance of the application and makes it harder to achieve minimum tension between the nodes. Switching to NJ mode does not affect the placement of the nodes, but determines the branching order of the network. It is possible to remove expression libraries or to add them to the analyzed set interactively, while the viewer automatically adjusts the diagram. The underlying Java3D API allows manipulating the graph in 3D coordinates, so the user has the ability to pan, rotate or zoom in/out. The viewer uses filenames as labels, which are shown on the selection list, as well as on the graph next to the nodes. Alternatively, the 'Description' option allows specification of an alternative description, which may be read from the header of the files with expression data.

**Figure 2:**
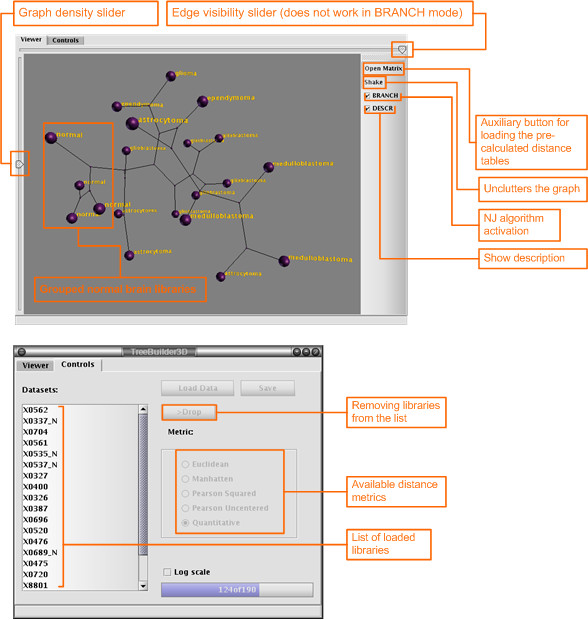
**visualization of SAGE data for normal and cancerous brain tissue with TreeBuilder3D**. **Above**: the viewer panel with SAGE data, visualized in 3D. The user can control the density of the graph (with the slider on the left) and visibility of the edges, which are shorter than the threshold (with the top slider). Additional buttons on the right side of the panel are explained on the diagram. It is also possible to pan/zoom/rotate using a 3-button mouse. **Below**: the control interface panel. A typical session starts with opening files using "Load Data" button and picking the desired distance metric. After that a 3D diagram is generated and user can start to overview the structure, adding/dropping libraries, where desired.

Figure 2 shows an example display of analysis done for publicly available SAGE data. The analysis was performed using SAGE data for 20 brain libraries, downloaded from the Gene Expression Omnibus Website (contributed by the laboratory of Dr. Riggins, John Hopkins University). All libraries had a short description of their clinical source, so it was possible to make some conclusions about quality of the generated diagram. Among the analyzed SAGE libraries 4 were prepared from normal brain tissue, while the other 16 libraries were prepared from different tumor samples. Although the brain tissue type for the libraries in the set were very heterogeneous, there was a distinct separation of normal and cancer libraries on the diagram. All normal libraries were directly connected and closely positioned on the graph, which shows that this approach to visualization can be useful for extracting order from SAGE data and organizing it into a meaningful structure. Files with microarray data, loaded from GEO website, can also be visualized in TreeBuilder3D.

## Conclusion

TreeBuilder3D provides a simple, comprehensible view of relationships in a hierarchy when applied to analysis of large-scale gene expression data generated by methods such as SAGE. An advantage of representing relationships between gene expression profiles in a three dimensional tree diagram is that it allows the visualization of subtleties in internodal relationships that could be overlooked due to the constraints imposed by a two-dimensional tree diagram. TreeBuilder3D allows the display of analyzed data in a more natural and intuitive way.

## Availability and requirements

The program is written in Java and runs on Windows or Linux platforms. In addition to compiled Java executable and documentation, we provide the source code for TreeBuilder3D under the terms of the General Public License (GPL) on our web site [3] (also see Additional file 1). TreeBuilder3D requires Sun's JRE 1.4 or later (downloadable from ), as well as Java3D package version 1.3.1, which is available for download at . Due to the limitations of the Java3D API, currently there is no support for Mac OS. Further information may be requested via e-mail to the corresponding author pruzanov@bcgsc.ca.

## Authors' contributions

PR and SJMJ developed the main ideas and methodology; PR did the coding; SJMJ provided feedback and coordination of the project. SJMJ and PR both read and approved the final manuscript.

## Supplementary Material

Additional File 1 contains the compiled Java executable, documentation and the source code for TreeBuilder3D. Example data sets are also included for the testing purposes. Click here for file
